# Deprescribing in Frail Older People: A Randomised Controlled Trial

**DOI:** 10.1371/journal.pone.0149984

**Published:** 2016-03-04

**Authors:** Kathleen Potter, Leon Flicker, Amy Page, Christopher Etherton-Beer

**Affiliations:** 1 School of Medicine and Pharmacology, University of Western Australia, Perth, Western Australia, Australia; 2 Department of Geriatric Medicine, Royal Perth Hospital, Perth, Western Australia, Australia; 3 Western Australian Centre for Health and Ageing, University of Western Australia, Perth, Western Australia, Australia; University of Glasgow, UNITED KINGDOM

## Abstract

**Objectives:**

Deprescribing has been proposed as a way to reduce polypharmacy in frail older people. We aimed to reduce the number of medicines consumed by people living in residential aged care facilities (RACF). Secondary objectives were to explore the effect of deprescribing on survival, falls, fractures, hospital admissions, cognitive, physical, and bowel function, quality of life, and sleep.

**Methods:**

Ninety-five people aged over 65 years living in four RACF in rural mid-west Western Australia were randomised in an open study. The intervention group (n = 47) received a deprescribing intervention, the planned cessation of non-beneficial medicines. The control group (n = 48) received usual care. Participants were monitored for twelve months from randomisation. Primary outcome was change in the mean number of unique regular medicines. All outcomes were assessed at baseline, six, and twelve months.

**Results:**

Study participants had a mean age of 84.3±6.9 years and 52% were female. Intervention group participants consumed 9.6±5.0 and control group participants consumed 9.5±3.6 unique regular medicines at baseline. Of the 348 medicines targeted for deprescribing (7.4±3.8 per person, 78% of regular medicines), 207 medicines (4.4±3.4 per person, 59% of targeted medicines) were successfully discontinued. The mean change in number of regular medicines at 12 months was -1.9±4.1 in intervention group participants and +0.1±3.5 in control group participants (estimated difference 2.0±0.9, 95%CI 0.08, 3.8, p = 0.04). Twelve intervention participants and 19 control participants died within 12 months of randomisation (26% versus 40% mortality, p = 0.16, HR 0.60, 95%CI 0.30 to 1.22) There were no significant differences between groups in other secondary outcomes. The main limitations of this study were the open design and small participant numbers.

**Conclusions:**

Deprescribing reduced the number of regular medicines consumed by frail older people living in residential care with no significant adverse effects on survival or other clinical outcomes.

**Trial Registration:**

Australian New Zealand Clinical Trials Registry ACTRN12611000370909

## Introduction

People consume an increasing number of medicines as they age.[1,2] In Australia, two-thirds of community dwelling adults aged 75 years and older are exposed to polypharmacy (the regular consumption of five or more medicines) and one in five take more than nine medicines daily.[3] Other developed countries report similar levels of medicine use.[1,4,5] People living in residential aged care facilities (RACF), particularly those with dementia, experience higher rates of polypharmacy than their community-dwelling peers. Approximately 90% of people living in Australian RACFs are prescribed five or more regular medicines and residents consume seven to ten medicines daily.[6–8]

The benefits of many medicines in frail older people are unquantified. Twenty-five to fifty per cent of clinical trials have a specific upper age limit and approximately 80% of clinical trials exclude people with co-morbidities. [9,10] Treatment guidelines based on such trials are often extrapolated to people who live in RACF despite an absence of evidence for benefit.[11] By contrast, the risks from many medicines in older people are well established. Older people are at high risk of adverse drug effects and toxicity due to reduced renal and liver function and age-related changes in physiological reserve, body composition, and cellular metabolism.[12] In frail older people the number needed to treat for some medicines is greater than the number needed to harm. [13–15]

Differentiating between the adverse effects of polypharmacy and the adverse effects of the co-morbidities targeted for treatment is difficult, but observational data suggest that polypharmacy independently increases the risk of frailty, falling, and hospital admission.[16,17] The more medicines an individual takes, the greater their risk of experiencing an adverse drug reaction, a drug-drug interaction, a drug-disease interaction, cascade prescribing, non-adherence, and drug errors (wrong drug, wrong dose, missed doses, erroneous dosing frequency).[18–21] Older people exposed to polypharmacy are not only at risk of harm from some of their medicines, they are also less likely to receive medicines that could help them. [6,22]

The cure for polypharmacy appears simple, doctors should prescribe and patients consume fewer medicines. Effecting this cure is not straight-forward. There are many barriers to reducing polypharmacy in practice, not least a lack of confidence on the part of doctors about when and how to cease medicines. [23,24] Doctors receive a great deal of information about the indications for starting medicines but very little guidance on when and how to stop them. In the absence of evidence to guide decision-making, doctors may feel it is simpler and safer to continue prescribing medicines than to discontinue them.[25,26]

Several randomised controlled trials in frail older people have investigated the effects of deprescribing, the planned cessation of non-beneficial medicines.[27–29] Deprescribing appears to reduce inappropriate medicine use but the effect on clinical endpoints such as hospital admissions and survival remains uncertain.

The primary objective of this study was to determine whether deprescribing would reduce the total number of medicines taken by frail older people living in RACF. Secondary objectives were to explore the effect of deprescribing on survival, falls, fractures, hospital admissions, cognitive, physical, and bowel function, quality of life, and sleep.

## Materials and Methods

### Ethics

This study obtained ethics approval from the University of Western Australia Human Research Ethics committee (RA/4/1/4517) and the WA Country Health Service Board Research Ethics Committee (ID 2011:21). Written informed consent was obtained from all competent participants. Written informed consent for the participation of people who were not competent was sought from the next of kin (NOK) or legal guardian. The study was conducted in accordance with the principles expressed in the Declaration of Helsinki.

### Design

Participants were randomised to an intervention (deprescribing) or control group (usual care) in a 1:1 ratio in an open trial with a parallel design.

### Participants

Every person living in an RACF in Geraldton, Western Australia (population ~40 500, 3 RACF, 241 beds) and Dongara, Western Australia (population ~3 800, 1 RACF, 6 beds) between July 2011 and December 2013 was screened for inclusion in the study. Residents were eligible to participate if they were aged 65 years or older. Residents were excluded if they were taking no regular medicines, were in the final terminal stages of an illness, or if their usual general practitioner (GP) or the RACF nurse manager did not agree to their participation.

### Intervention

The intervention was an individualised medicine review followed by the planned cessation of non-beneficial medicines. Both groups received a medicine review but only intervention group participants were deprescribed. Both groups received regular monitoring visits from KP (a general practitioner) and usual care from their own GPs. The intention of deprescribing was to reduce the total number of unique medicines consumed by intervention group participants.

#### Medicine review

All RACF residents received their regular oral medicines from a personal blister pack supplied weekly from a community pharmacy. A nurse administered each medicine dose, including regular medicines given by a non-oral route (eye drops, ear drops, skin lotions, ointments, analgesic patches, and insulin injections), and signed a pharmacy-generated administration record indicating if the medicine had been given, withheld, or declined. Records of the date, time, and dose of all pro re nata (PRN) and nurse-initiated medicines were also kept.

KP compiled a list of all medicines used by each participant at baseline from the drug chart and the most recent administration record. Discrepancies between the drug chart and the administration record were resolved by inspecting the blister pack. Participants were asked to list all self-administered medicines. The generic name, dose, frequency, and route of administration of all medicines available for use by the participant were recorded. The number of doses of each PRN, nurse-initiated, and self-administered medicines consumed in the preceding month were recorded.

A medicine-focused clinical history was compiled from the RACF medical records and progress notes by KP. She also interviewed each participant, their NOK, the nurse manager, and/or treating doctor. An indication was recorded for each medicine. Relevant co-morbidities, contraindications, and possible adverse effects were recorded; for example, regular nose bleeds or unexplained iron deficiency anaemia in a resident taking aspirin or anticoagulants, recurrent falls or postural hypotension in a resident on antihypertensive agents. Participants and nursing staff were asked specifically about possible medicine side effects, for example a dry mouth or urinary dysfunction in people taking anticholinergic medicines, ankle oedema and constipation in people taking calcium-channel blockers. Participants were asked if they were still experiencing symptoms that were the intended target of specific treatments, for example reflux symptoms in people taking a proton-pump inhibitor, joint pain in people taking paracetamol or a non-steroidal anti-inflammatory. All participants were asked whether they experienced any of the following common medication side effect symptoms: nausea, constipation, diarrhoea, abdominal pains, dry mouth, dizziness, headaches, insomnia, skin rash or itch, cough, ankle swelling, and dry eyes. Frequency (not during past month, less than once per week, once or more per week, daily or almost daily) and severity (causing mild, moderate, or severe distress) were recorded for any symptoms reported. KP examined all participants for cachexia, muscle wasting, tremor, rashes, cough, dyspnoea, heart murmurs, basal crepitation, ankle oedema, and for signs of discomfort, depression, anxiety, agitation, and/or confusion.

One experienced registered nurse (RN) performed baseline assessments. KP repeated the assessments on the first five participants after one week and results were compared to ensure consistency. The baseline assessments consisted of blood pressure (three sitting or lying blood pressures taken at five minute intervals followed by a standing or sitting blood pressure), tibial length, weight (most recent weight recorded by the RACF), symptom check list (described above), and bowel function assessed over the previous 14 days from the most recent bowel chart (number of bowel motions, any episodes of faecal incontinence, number of episodes of faecal incontinence, number of days with no bowel motion). The RN used validated questionnaires to assess cognitive function,[30] (30) sleep quality, quality of life and self-reported general health.[31] (31) She also interviewed a carer to assess sleep quality and physical function.

#### Deprescribing

**Fig 1:**
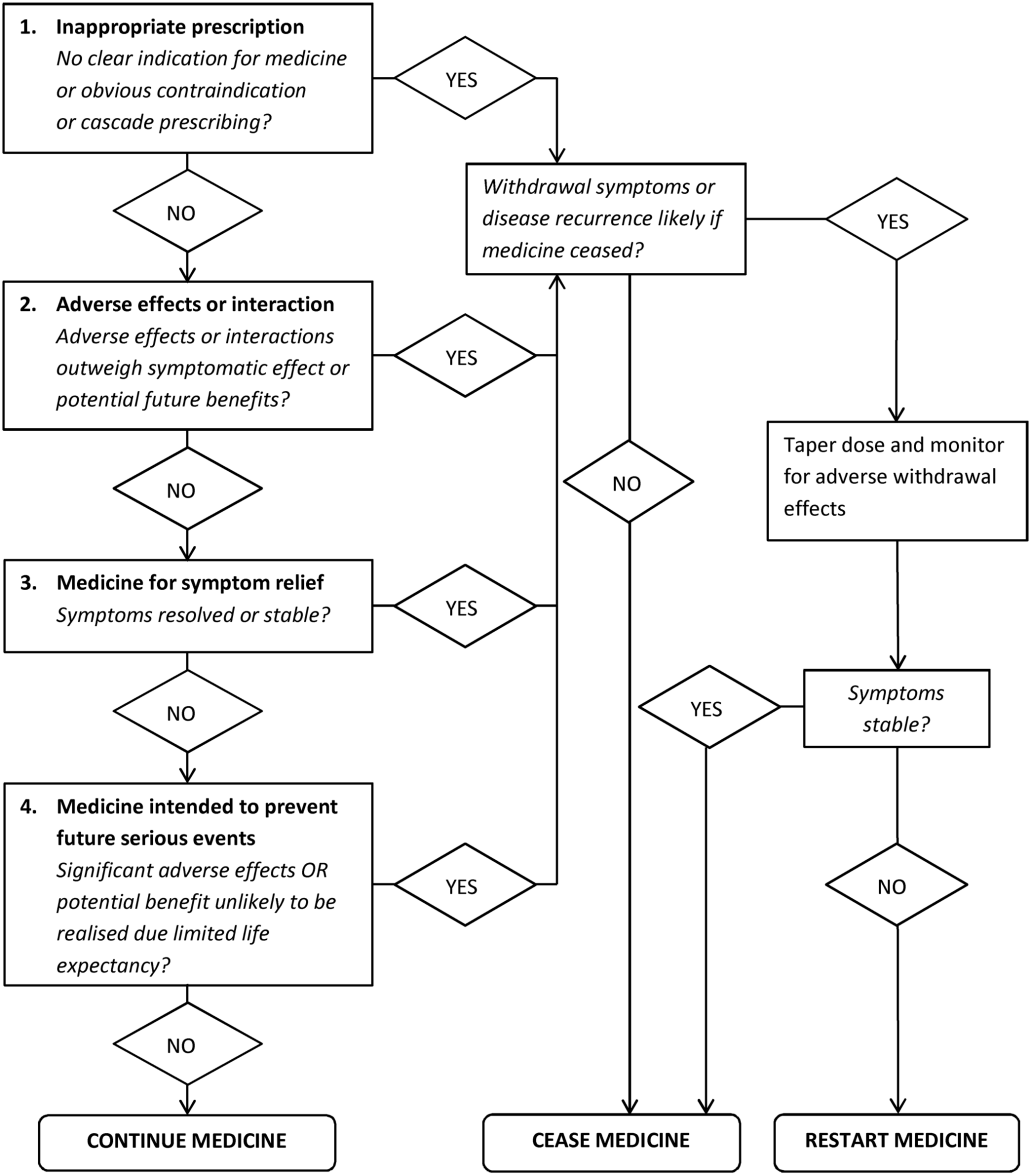
Deprescribing Algorithm.

Two investigators (KP, a general practitioner, and CEB, a geriatrician/clinical pharmacologist) independently identified deprescribing targets using a list of potentially inappropriate medicines (S1 Table). [32–37] They used baseline data (clinical history and examination findings, baseline MMSE and MBI scores, BP measurements, weight, bowel function, and side-effects questionnaire)to test each target medicine against four deprescribing criteria defined in Fig 1.

Medicines intended for symptom relief were considered for deprescribing if symptoms were stable according to pre-defined criteria (S2 Table). The investigators determined which medicines required dose tapering prior to cessation and planned a cessation order. First they targeted medicines causing active harm to the participant (contraindicated medicines, toxic medicines with no clear indication, medicines causing significant adverse effects). Next they targeted medicines unlikely to be benefitting the participant and unlikely to cause adverse withdrawal effects (eg. multivitamins in people with an adequate nutritional intake, aspirin and statins in people with no history of vascular disease). Then they targeted medicines with possible adverse effects and a high potential for adverse withdrawal reactions or rebound symptoms (eg. benzodiazapines, anti-reflux medicines, antihypertensives, antidepressants). Finally they targeted low risk medicines intended for symptomatic relief where symptoms were stable (eg. paracetamol, sorbolene cream, osmotic laxatives).

When both investigators had completed a medicine withdrawal plan for each participant (an individualised list of target medicines with dose tapering recommendations and cessation order), they compared the plans and discussed any discrepancies to generate a final consensus medicine withdrawal plan. Group allocation was revealed after the consensus plan was finalised.

GPs were advised in writing of group allocation and asked to proceed according to their clinical judgement if they wished to start or stop any medicine during the trial period. They were given the individualised medicine withdrawal plans for each intervention group participant and asked to identify any medicines that should not be stopped. All participants and/or NOK were advised of group allocation. KP discussed the medicine withdrawal plan with intervention group participants and/or NOK and removed from the plan any medicines they declined to cease. Control group participants and/or NOK were advised to discuss any medicine-related concerns with their own GP.

The medicine withdrawal plan, amended to reflect changes requested by participant, NOK, or GP, was implemented over several months. In the first five participants, individual medicines were ceased sequentially with two weeks between withdrawals or dose changes. The study protocol was subsequently amended to allow the withdrawal of up to three medicines simultaneously, provided the medicines were unlikely to cause adverse withdrawal effects or that any adverse withdrawal effects or symptom recurrence would be attributable to a single culprit medicine. Before ceasing or reducing the dose of medicines likely to cause adverse withdrawal effects KP discussed potential adverse effects with the participant and/or carers. If an RN did not want a medicine stopped, cessation was not attempted. Dose reductions and cessation orders were faxed or emailed to the pharmacist and GP. Expected cessation date, dose changes, and a description of possible adverse withdrawal effects were recorded in the participant’s notes.

KP reviewed participants weekly during deprescribing. She measured blood pressure, elicited any symptoms related to the medicine changes and examined the participant for signs of distress and/or adverse medicine withdrawal effects. She inspected blister packs to confirm dose change or cessation, checked progress notes for reports of adverse withdrawal effects or symptom recurrence, and asked carers if they had observed any changes in the participant. If withdrawal effects or signs or symptoms of disease recurrence were confirmed, the deprescribed medicine was restarted and the GP informed of the withdrawal failure. All participants were reviewed on each RACF visit to ensure control group participants were visited as frequently as intervention group participants. Control group participants had blood pressure measured and were asked if they had any concerns at each visit. Any significant issues in control group participants were reported to nursing staff for appropriate follow-up.

### Outcomes

#### Primary outcome

The primary outcome was the mean change in the number of unique regular medicines consumed by participants at 12 months post-randomisation. The total number of regular medicines was comprised of all regular medicines, any PRN, nurse-initiated or self-administered medicine that had been used more frequently than once per week during the preceding 3 month period (>13 doses), and any short term medicine (eg. antibiotics, topical steroids) being used by the participant on the date of the 12 month follow-up. A medicine was defined as any prescribed, non-prescribed, complementary, or alternative medicine. Medicines were classified using the Anatomical Therapeutic Classification (ATC) system. Medicines with a single active ingredient were counted as a one medicine. The active ingredients of combination products were counted as individual medicines if available in a similar dose and form as a single medicine (Australian Medicines Handbook [38]) and an ATC code for the intended therapeutic purpose existed. Alternative and complementary medicines were allocated an ATC code that most accurately reflected their intended therapeutic purpose. Multivitamins, mineral supplements, topical treatments, eye drops, and haemorrhoid creams were counted as a single medicine unless the criteria for combination products were met.

### Secondary outcomes

- Survival at 12 months post-randomisation.
- Proportion of participants experiencing a fall or non-vertebral fracture (confirmed by radiological assessment). A fall was defined as anything reported as a fall in the progress notes or any incident where a participant was found kneeling, sitting, or lying on the floor by NOK or an RACF staff member.
- Proportion of participants experiencing an unplanned hospital admission or out-of-hours GP visit (defined as an unscheduled GP visit before 8am or after 6pm on a weekday or on a Saturday or Sunday or an ambulance call where the participant was not transported to hospital).
- Change in the mean number of medicines consumed by participants at three, six and nine months.

The outcomes listed above were assessed by KP who was aware of treatment allocation. She recorded these data from the RACF progress notes and medicine charts every three months. The outcomes listed below were assessed by one RN who was blind to treatment allocation. These outcomes were assessed using validated questionnaires (unless otherwise specified) at baseline, six, and twelve months.

- Cognitive function assessed with the Mini Mental Status Examination (MMSE).[30]
- Physical function assessed by proxy with the Modified Barthel Index (MBI).[39]
- Bowel function assessed using the RACF bowel chart.
- Self-reported quality of life assessed with Quality of Life in Alzheimer’s Dementia (QOLAD).[40]
- Self-reported general health assessed with the EQ-5D.[31]
- Sleep quality assessed by proxy with the Neuropsychiatric Index—Nursing Home Version (NPI-NH) in all participants and self-assessed with the Pittsburgh Sleep Quality Index (PSQI) in participants with an MMSE score >23. [41,42]

### Sample size

This trial was designed to have adequate power to detect a clinically relevant change in the number of unique regular medicines at 12 months. A clinically relevant reduction in medicines was assumed to be between one and two regular medicines per person. A secondary objective was to explore the effect of deprescribing on other clinical outcomes. We planned to enrol 250 participants. This number would have given us adequate power to detect a mean change of ±1.34 in the number of medicines (α = 0.05 and 1-β = 0.8, SD 3.9). The total number of eligible RACF residents in Geraldton and Dongara during the recruitment period was 324 however, and we were unsuccessful in establishing a second recruitment site. Consequently, our participant numbers were too small to precisely estimate the effect of deprescribing on the secondary outcomes.

### Randomisation

The random allocation sequence was generated prospectively by a statistician who used a digital random number generator to create permuted blocks of 2, 4, 6, and 8. The group (A or B) was printed on a slip of paper and sealed in an opaque envelope by the statistician. Envelopes were labelled consecutively from 1 to 250. Participants were assigned an envelope in the order in which they were enrolled. The envelopes were opened to reveal allocation only after the medication review, medication withdrawal plan, and baseline assessments were complete.

### Blinding

The RN who performed baseline and secondary outcome assessments was blind to group allocation. The primary investigator (KP), community pharmacists, GPs, participants, NOK, and RACF nurses and carers were all aware of group allocation.

### Statistical methods

Data were analysed on an intention-to-treat basis with per protocol statistical tests. SPSS v.22 was used for statistical analyses. The primary outcome of change in the mean number of unique regular medicines at one year was assessed with an independent t-test. Mortality was assessed with Kaplan-Meier survival curves and the Cox proportional hazards model was used to compare survival in the intervention and control groups. Fisher’s exact test was used to compare the proportion of participants in each group who had experienced an after-hours GP visit, a hospital admission, a fall, or a fracture at six and twelve months post-enrolment. The MMSE, MBI, QOLAD, EQ-5D, PQSI and NPI-NH sleep scores at six and twelve months were compared using generalised linear models adjusted for age, sex, and number of regular medicines at baseline. The models used were random effects linear models with maximum likelihood estimation for the EQ-5D, MMSE, QOLAD and PSQI scores and a random effects negative binomial model for the MBI and NPI-NH scores. These models included a test for group effect, time effect and a time*group interaction. P-values for all tests are reported and p-values of less than 0.05 are considered significant. The study was inadequately powered to assess secondary outcomes. The secondary analyses should be viewed as hypothesis-generating and the p-values interpreted with caution. All data are reported as mean ± standard deviation unless otherwise stated.

## Results

### Participant recruitment

**Fig 2:**
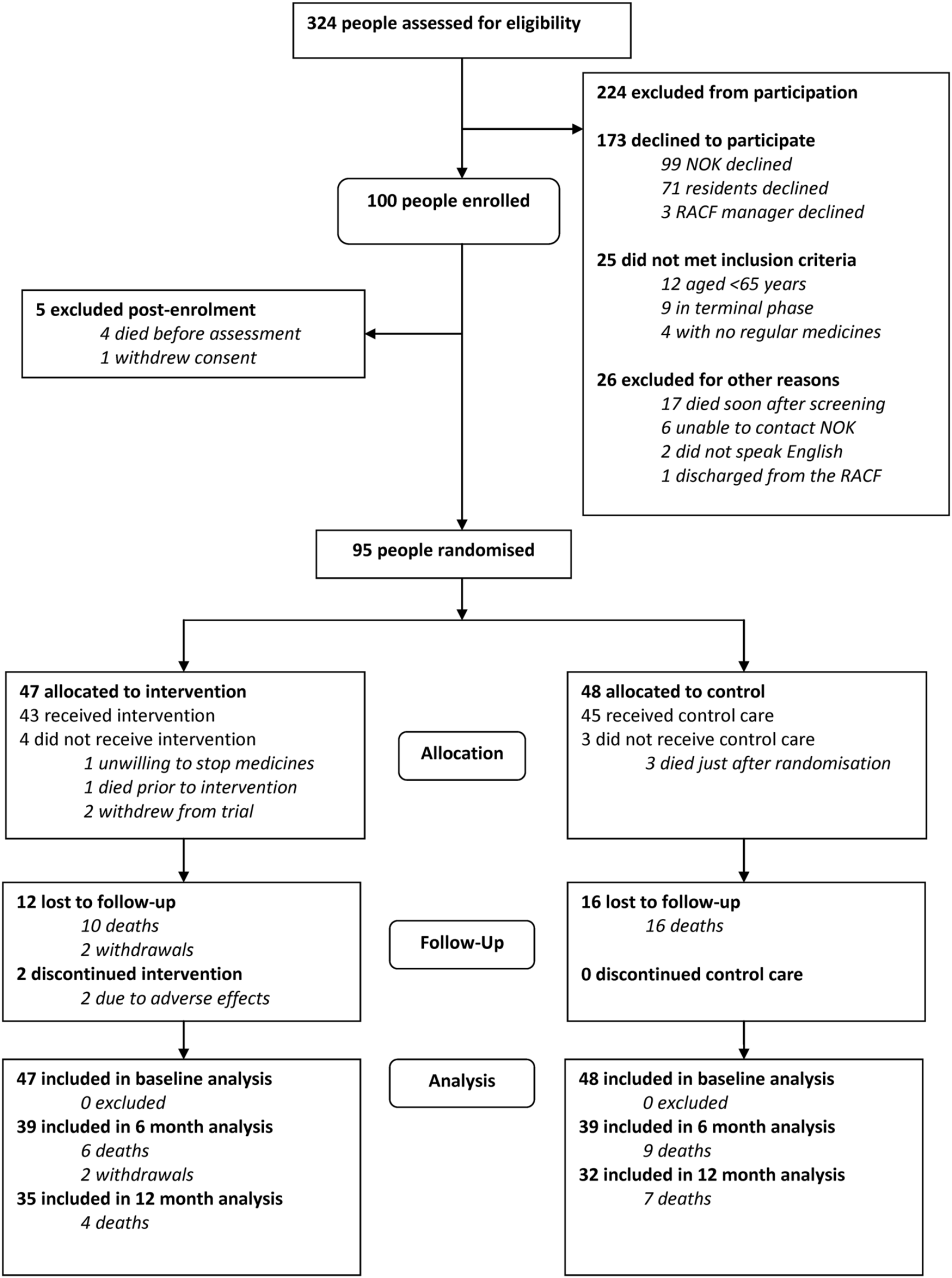
Recruitment and Participation.

Every individual living in an RACF in Dongara or Geraldton between 19^th^ July 2011 and 12^th^ November 2013 was screened for eligibility (n = 324). Approximately one third of the eligible population were enrolled in the trial (n = 100, 31%). Three quarters of the participants (n = 75) had cognitive impairment (MMSE score <24) and their NOK were required to provide formal consent to participation. All participants were followed for twelve months from randomisation or until death. Two participants (1 control, 1 intervention) died less than 72 hours before their final assessment was due and one participant (control) was moribund on the due date for his final assessment. The medicines, adverse outcomes, and bowel function data from these participants were included in the 12 month analyses but other secondary outcomes were not collected. There was no difference between the groups in the frequency of review visits during the study period (intervention group 16 ± 7 review visits, control group 15 ± 6 review visits, estimated difference 0.91, 95% CI -1.88, 3.69, p = 0.52). Fig 2 shows recruitment and participation.

### Baseline data

Table 1 shows baseline demographic data, clinical characteristics, and medicines data for each group. A greater proportion of study participants were male than the screened population (0.48 versus 0.35, p = 0.02), but the mean age of participants and eligible non-participants was similar (84 ± 7 years versus 85 ± 7, p = 0.32). Control group participants had a lower mean systolic blood pressure than intervention group participants at baseline (standing BP of 114 mmHg versus 135mmHg, p<0.001). The reason for this difference is unclear. Similar proportions of control group and intervention group participants were taking at least one antihypertensive agent at baseline (n = 33, 69% versus n = 27, 57%, p = 0.20) and the mean number of antihypertensive agents did not differ significantly between groups (2.2 ± 1.2 versus 1.9 ± 1.2, p = 0.29). A similar proportion of both groups had a diagnosis of hypertension recorded in their RACF medical records at baseline (control n = 32 (67%) and intervention n = 30 (64%)). S3 Table shows frequencies of the most common medical diagnoses recorded in participant RACF records at baseline. The only significant differences between groups at baseline were in gastro-oesophageal reflux disease (control n = 28, 58% and intervention n = 17, 36%, p = 0.04) and chronic kidney disease (n = 2, 4% and n = 8, 17%, p = 0.05). The apparent differences in ischemic heart disease (n = 14, 29% and n = 6, 13%) and fractures (n = 8, 17% and n = 16, 34%) were not significant. However RACF medical records are not a complete clinical record and we are thus unable to verify the accuracy or completeness of the listed diagnoses.

**Table 1:** Baseline demographic and clinical data.

	Group	
	Intervention (n = 47)	Control (n = 48)
Gender (male, n, %)^a^	21 (45)	25 (52)
Age (years)^b^	84 (6)	84 (8)
Weight (kg)	65 (17)	69 (15)
Tibial length (cm)	37 (3)	37 (3)
BP systolic (seated or lying, mmHg)	131 (21)	123 (21)
BP systolic (standing or sitting, mmHg)	135 (22)	114 (19)
BP diastolic (seated or lying, mmHg)	69 (13)	66 (10)
BP diastolic (standing or sitting, mmHg)	75 (17)	66 (16)
Heart rate (seated or lying, bpm)	71 (13)	72 (12)
Heart rate (standing or sitting, bpm)	78 (14)	83 (16)
MMSE (/30)	15 (10)	13 (8)
MBI (/100)	48 (35)	45 (32)
QOLAD (/52, n = 30, n = 30)	33 (6)	32 (6)
EQ-5D (/100, n = 28, n = 27)	71 (15)	63 (19)
NPI-NH sleep section (/12, n = 39, n = 45)	3 (4)	1 (3)
PSQI (/21, n = 17, n = 8)	5 (3)	5 (3)
Number of bowel motions	10 (6)	11 (6)
Any episodes of faecal incontinence (n, %)	19 (40)	16 (33)
Number of episodes of faecal incontinence	9 (9)	10 (7)
Number of days bowels not open	6 (3)	5(3)
Regular medicines	9.6 (5.0)	9.5 (3.6)
PRN and nurse-initiated medicines	4.3 (3.1)	3.5 (2.2)
PRN used	1.8 (1.7)	1.2 (1.4)
PRN not used	2.5 (2.4)	2.4 (2.0)
Target medicines for deprescribing	7.4 (3.8)	7.9 (3.7)

Numbers are mean (SD) or n (%).

^a^35% of screened residents were male.

^b^Mean age of eligible non-participants was 85 ± 7 years.

BP, blood pressure; MMSE, Mini-Mental Examination Score; MBI, Modified Barthel Index; QOLAD, Quality of Life in Alzheimer's Dementia; NPI-NH, Neuropsychiatric Index—Nursing Home version; PSQI, Pittsburg Sleep Quality Index; EQ-5D, VAS score; PRN, pro re nata (as needed).

All bowel data recorded from bowel charts over the 14 days immediately prior to the assessment date. “Number of episodes of faecal incontinence” is mean number of episodes in the individuals with at least once episode of incontinence. “Regular medicines” is the sum of all regular medicines AND all PRN, nurse-initiated, or self-administered medicines used more frequently than once per week. “PRN used” is the number of PRN or nurse-initiated medicines used at least once in the month prior to the baseline assessment. “PRN not used” is the number of PRN or nurse-initiated medicines not used in the month prior to the baseline assessment.

### Primary outcome

**Fig 3:**
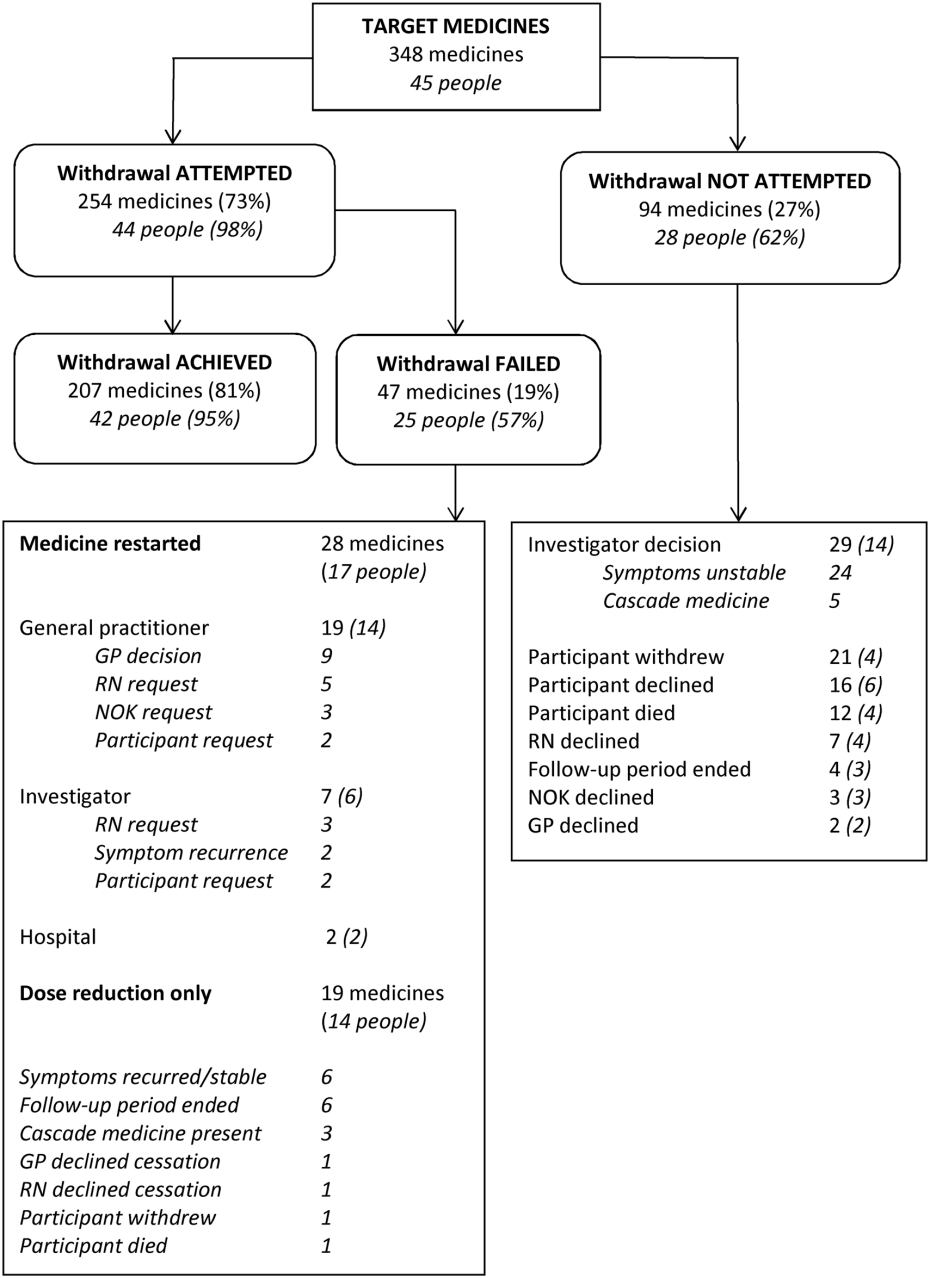
Deprescribing Outcomes. “Withdrawal Achieved” means medicine ceased or changed to PRN used less frequently than once per week at the last follow-up assessment point; RN, registered nurse; NOK, next of kin; GP, general practitioner. Percentages for “Withdrawal Failed” and “Withdrawal Achieved” are calculated as a percentage of the “Withdrawal Attempted” total. Italic numbers in () in the two large explanatory boxes refer to number of people.

Fig 3 shows deprescribing outcomes in the intervention group. Two intervention group participants had no medicines suitable for cessation. At least one medicine was ceased in 89% of participants (42/47 participants) and 4.4 ± 3.4 medicines per person were successfully ceased. Withdrawal was either not attempted or failed for 41% of the medicines selected for deprescribing (n = 141/348 medicines). The main reasons for no withdrawal attempt were participant death or withdrawal from the study (n = 33 medicines, 9% of targets), investigator decision against cessation (n = 29 medicines, 8% of targets), and participant decision to continue a medicine (n = 16 medicines, 5% of targets). Withdrawal failures were due to the medicine being restarted (n = 28 medicines, 8% of targets) or dose reduction without cessation (n = 19 medicines, 5% of targets).

Table 2 shows withdrawal success rates for the most frequently deprescribed medicines (targeted in ten or more participants). Medicines with highest withdrawal success rates were bisphosphonates, aspirin, iron supplements (8/9, 89%), angiotensin II antagonists, vitamin and mineral supplements, and statins. Medicines with the lowest withdrawal success rates were anti-epileptics (2/8, 25%), laxatives, analgesics, antidepressants, proton-pump inhibitors, and benzodiazepines. The full list of medicines targeted for deprescribing is reported in S4 Table.

**Table 2:** Withdrawal success rates for medicines deprescribed in ten or more participants.

Medicine Class (ATC code)	Deprescribing target (n)	Withdrawal attempted n (%)	Successful withdrawal n(%)	Overall withdrawal success (%)
**Laxatives (A06)**	**36**	**19 (53)**	**10 (53)**	**28**
*Coloxyl and Senna*^a^	*13*	*7 (54)*	*4 (57)*	*31*
*Movicol (A06AD65)*	*8*	*5 (63)*	*2 (40)*	*25*
*Others*	*2*	*0 (0)*	*0 (0)*	*0*
**Analgesics (N02)**	**32**	**15 (47)**	**11 (73)**	**34**
*Paracetamol (N02BE01)*	*22*	*11 (50)*	*7 (64)*	*32*
*Opioids (N02A)*	*10*	*4 (40)*	*4 (100)*	*40*
**Psychoanaleptics (N06)**	**25**	**20 (80)**	**10 (50)**	**40**
*Antidepressants (N06A)*	*22*	*17 (77)*	*8 (47)*	*36*
*Anti-dementia drugs (N06D)*	*3*	*3 (100)*	*2 (67)*	*67*
**Antithrombotic agents (B01)**	**24**	**23 (96)**	**21 (91)**	**88**
*Aspirin (B01AC06)*	*18*	*18 (100)*	*17 (94)*	*94*
*Others*	*6*	*5 (83)*	*4 (80)*	*67*
**Drugs for acid-related disorders (A02)**	**22**	**18 (82)**	**13 (72)**	**59**
*Proton pump inhibitors (A02BC)*	*19*	*15 (79)*	*10 (67)*	*53*
*H2 receptor antagonists (A02BA)*	*3*	*3 (100)*	*3 (100)*	*100*
**Vitamins (A11)**	**19**	**16 (84)**	**16 (100)**	**84**
*Vitamin D (A11CC)*	*12*	*12 (100)*	*12(100)*	*100*
*Vitamin C (A11GA)*	*3*	*3 (100)*	*3 (100)*	*100*
*Others*	*4*	*1 (25)*	*1(100)*	*25*
**Psycholeptics (N05)**	**17**	**12 (71)**	**8 (67)**	**47**
*Antipsychotics (N05A)*	*9*	*8 (89)*	*6 (75)*	*67*
*Hypnotics (N05CD)*	*5*	*2 (40)*	*1 (50)*	*20*
*Anxiolytics (N05BA)*	*3*	*2 (67)*	*1 (50)*	*33*
**Lipid modifying agents (C10)**	**17**	**16 (94)**	**14 (88)**	**82**
*HMG CoA reductase inhibitors (C10AA)*	*13*	*12 (92)*	*10 (83)*	*77*
*Omega-3-triglycerides (C10AX)*	*4*	*4 (100)*	*4 (100)*	*100*
**Drugs for obstructive airways disease (R03)**	**15**	**9 (60)**	**9 (100)**	**60**
*Beta-2-adrenoreceptor agonists (R03AC)*	*6*	*4 (67)*	*4 (100)*	*67*
*Fluticasone (R03BA05)*	*5*	*3 (60)*	*3 (100)*	*60*
*Tiotropium bromide (R03BB04)*	*4*	*2 (50)*	*2 (100)*	*50*
**Mineral supplements (A12)**	**15**	**12 (80)**	**12 (100)**	**80**
*Calcium (A12AA)*	*7*	*5 (71)*	*5 (100)*	*71*
*Magnesium (A12CC)*	*4*	*4 (100)*	*4 (100)*	*100*
*Potassium (A12BA)*	*3*	*3 (100)*	*3 (100)*	*100*
*Sodium (A12CA)*	*1*	*0 (0)*	*0 (0)*	*0*
**Drugs acting on the renin-angiotensin system (C09)**	**15**	**14 (93)**	**13 (93)**	**87**
*Angiotensin II antagonists (C09C)*	*9*	*9 (100)*	*9 (100)*	*100*
*ACE inhibitors (C09A)*	*6*	*5 (83)*	*4 (80)*	*67*
**Calcium channel blockers (C08)**	**12**	**12 (100)**	**8 (67)**	**67**
*Dihydropyridine derivatives (C08CA)*	*9*	*9 (100)*	*5 (56)*	*56*
*Benzothiazepine derivatives (C08DB)*	*2*	*2 (100)*	*2 (100)*	*100*
*Phenylalkylamine derivatives (C08DA)*	*1*	*1 (100)*	*1 (100)*	*100*
**Drugs for treating bone disease (M05)**	**11**	**10 (91)**	**10 (100)**	**91**
*Bisphosphonates (M05BA)*	*10*	*9 (90)*	*9 (100)*	*90*
*Strontium ralenate (M05BX)*	*1*	*1 (100)*	*1 (100)*	*100*
**Beta-blocking agents (C07A)**	**10**	**5 (50)**	**5 (100)**	**50**
Selective beta-blocking agents (C07AB)	10	5 (50)	5 (100)	50
**TOTAL**	**270**	**201 (74)**	**160 (80)**	**59**

^a^Coloxyl and senna counted as two separate medicines in Laxatives (A06), docusate sodium (A06AA02) and senna glycosides (A06AB56).

**Fig 4:**
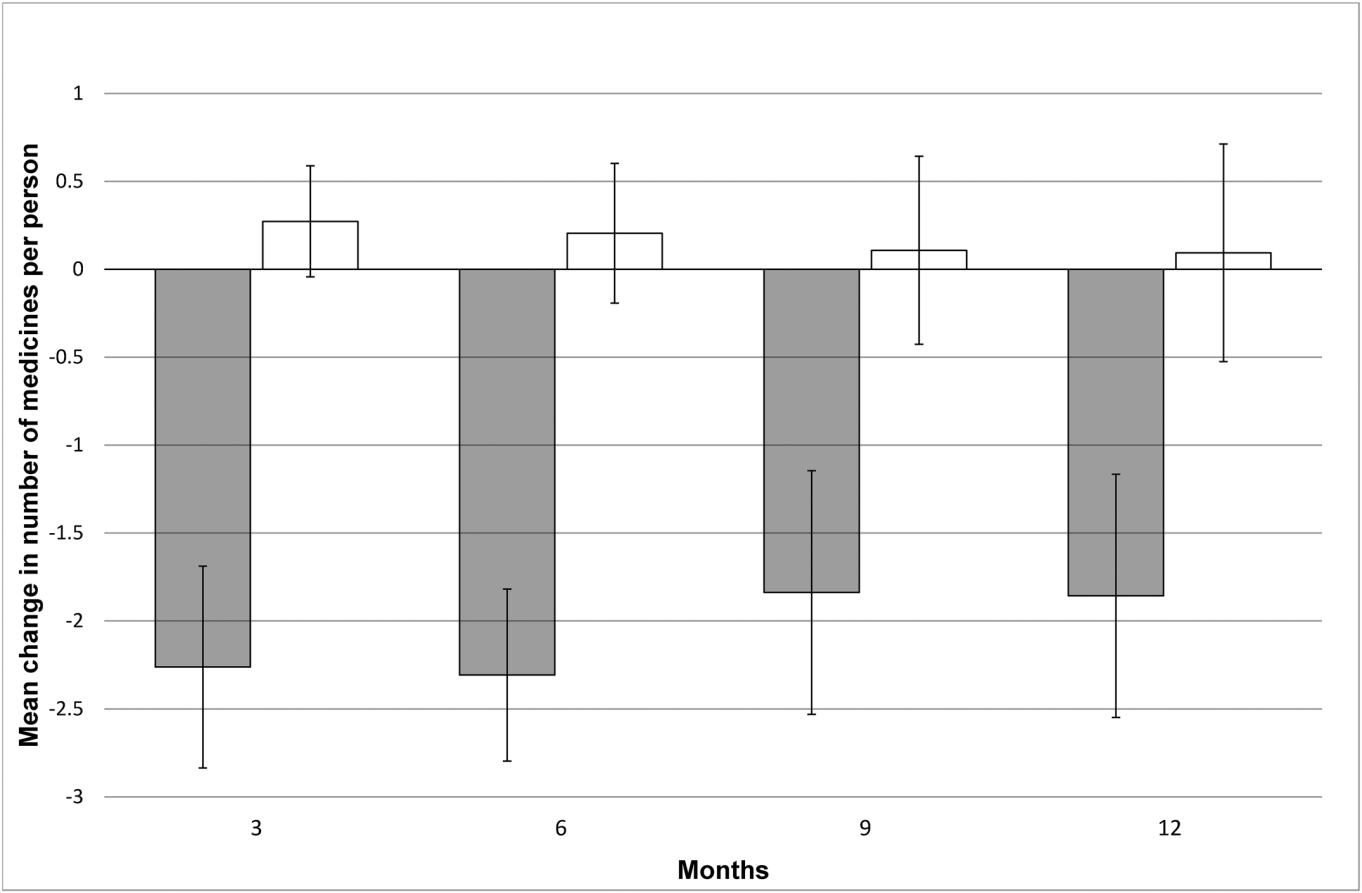
Change in the mean number of regular medicines per person. Closed bars are the intervention group. Open bars are the control group. Error bars are 1 SEM.

Fig 4 shows change in the mean number of unique regular medicines consumed by participants during the study period. At 12 months the mean change was -1.9 ± 4.1 in the intervention group and +0.1 ± 3.5 in the control group (estimated difference 2.0 ± 0.9, 95%CI 0.08, 3.8, t = 2.09, df = 65, p = 0.04). The maximal change in number of medicines occurred at six month post-randomisation (-2.3 ± 3.1 versus +0.2 ± 2.5, estimated difference 2.5, 95% CI 1.3, 3.8, t = 3.98, df 76, p<0.001) Although 4.4 ± 3.4 regular medicines per participant were successfully deprescribed (no longer consumed at the last follow-up assessment), many new medicines were started during the study period. Eighty per cent of surviving intervention participants and 72% of surviving control participants were taking at least one new regular medicine at 12 months (2.9 ± 2.0 new medicines in 28 participants versus 3.0 ± 2.1 in 23 participants, p = 0.85). The most commonly prescribed new medicines in the intervention group were laxatives (A06, n = 12), analgesics (N02, n = 8), eye drops (S01, n = 6), topical antifungal agents (D01, n = 5), and vitamins (A11, n = 5). Skin emollients (D02, n = 8), analgesics (N02, n = 7), inhaled agents for obstructive airways disease (R03, n = 6), and laxatives (A06, n = 5) were the most commonly initiated new medicines in the control group. The full list of new regular medicines prescribed for surviving participants at 12 months is reported in S5 Table.

### Secondary outcomes

Questionnaire scores are in Table 3. Bowel function data is in Table 4.

**Table 3:** Cognitive function, independence in ADLS, sleep quality, self-assessed quality of life, self-assessed general health.

Outcome	6 months				12 months					
	Intervention		Control		Intervention		Control		p (raw)	p (adj)
	Change	n	Change	n	Change	n	Change	n		
MMSE	-2 (5)	39	-1 (5)	39	-3 (5)	34	-2 (4)	30	0.54	0.60
MBI	-8 (19)	39	-7 (14)	38	-10 (17)	34	-11 (15)	30	0.76	0.76
QOLAD	-0.7 (4.4)	23	-0.2 (4.8)	22	-1.0 (4.3)	22	-1.0 (4.7)	15	0.94	0.91
EQ-5D	-11 (24)	20	1(29)	17	-11 (17)	20	7 (15)	12	0.25	0.35
NPI-NH	-0.4 (4.9)	34	-0.1 (2.7)	39	-0.1 (4.7)	28	-0.2(2.3)	30	0.98	0.95
PSQI	-1 (3)	13	0 (1)	4	0 (3)	9	-1 (2)	3	0.78	0.76

Values are the mean (SD) change in score at six months and twelve months.

MMSE, Mini-Mental State Examination; MBI, Modified Barthel Index; QOLAD, Quality of Life in Alzheimer’s Dementia; EQ-5D –VAS score; NPI-NH, Neuropsychiatric Index—Nursing Home Version; PQSI, Pittsburgh Sleep Quality Index.

For MMSE, MBI, QOLAD and EQ-5D, negative values represent worse cognitive function, physical function, quality of life, and general health respectively. For the PSQI and NPI-NH, negative values represent improved sleep quality.

P-values for MMSE, QOLAD, PQSI, and EQ-5D are from random effects linear models with maximum likelihood estimation. P-values for MBI and NPI-NH are from random effects negative binomial models. The p-values are for the between-group differences at 12 months. Adjusted p-values are from a model including age, sex, and number of regular medicines at baseline.

**Table 4:** Change in bowel function following deprescribing.

Bowel function*	6 months		p	12 months		p
	Intervention n = 39	Control n = 39		Intervention n = 34	Control n = 32	
Bowel motions	1.7 (7.0)	0.8 (3.8)	0.51	0.9 (3.7)	2.4 (6.3)	0.94
Any episode of faecal incontinence (n, %)	18 (46)	21 (54)	0.65	15 (44)	20 (63)	0.15
Episodes of faecal incontinence	3.9 (8.9)	2.9 (6.6)	0.65	3.6 (7.6)	2.8 (10.4)	0.77
Days with no bowel motion	-1.0 (3.7)	-0.4 (2.7)	0.53	-1.6 (3.9)	-1.4 (3.2)	0.86

Values are mean change (SD) from baseline or n (%).

*All data were recorded from the RACF bowel chart over the 14 day period immediately prior to the assessment date. Episodes of faecal incontinence represent the mean change in number of episodes of faecal incontinence in the individuals with at least once episode of incontinence.

P-values are from a 2 sample t-test or Fisher’s exact test for proportions.

### Adverse outcomes

Four serious vascular events occurred during the study period. Three events were in control group participants (one ischemic cerebrovascular event confirmed by CT and two acute coronary events, a NSTEMI with ECG changes and an episode of unstable angina) and one event was in an intervention group participant (acute coronary syndrome, an elevated troponin rise only, no ECG changes). Two intervention group participants experienced significant adverse medicine withdrawal reactions. One man was admitted to hospital in October 2012 with symptomatic rapid atrial fibrillation following cessation of his amiodarone in May 2012. The second man became agitated when his oxazepam dose was reduced from twice daily dosing to once daily dosing. Both participants withdrew from the study after these events but their data were included in analyses. Other adverse outcomes are reported in Table 5.

**Table 5:** Adverse outcomes.

Outcome	Intervention (n = 45)		Control (n = 48)		p
	Proportion (95%CI)	Number of participants (number of events)	Proportion (95%CI)	Number of participants (number of events)	
Fall	0.56 (0.42, 0.69)	25 (221)	0.65 (0.50, 0.77)	31 (142)	0.40
Fracture	0.07 (0.02, 0.19)	3 (3)	0.04 (0.004, 0.15)	2 (2)	0.67
GP attendance	0.22 (0.12, 0.36)	10 (18)	0.10 (0.04, 0.23)	5 (10)	0.16
Call to GP	0.53 (0.39, 0.67)	24 (83)	0.60 (0.46, 0.67)	29 (71)	0.53
Hospital admission	0.51 (0.37, 0.61)	23 (43)	0.50 (0.36, 0.63)	24 (44)	0.99

P-values are from a Fisher’s exact test.

95% confidence intervals were calculated by the modified Wald method.

Events are reported as the proportion of participants experiencing at least one event during the trial period.

Fall; any witnessed event recorded as a fall in the RACF progress notes, any unwitnessed incident where a resident was found sitting, lying, or kneeling on the floor, fracture; a new, non-vertebral fracture confirmed by radiological investigation, GP visit; unscheduled visit by GP or ambulance attendance without hospital transfer, GP call; phone call to a GP or emergency department requiring a response (excludes faxes, calls to a GP practice not requiring a doctor to respond, and routine phone notifications requiring no response).

### Mortality

**Fig 5:**
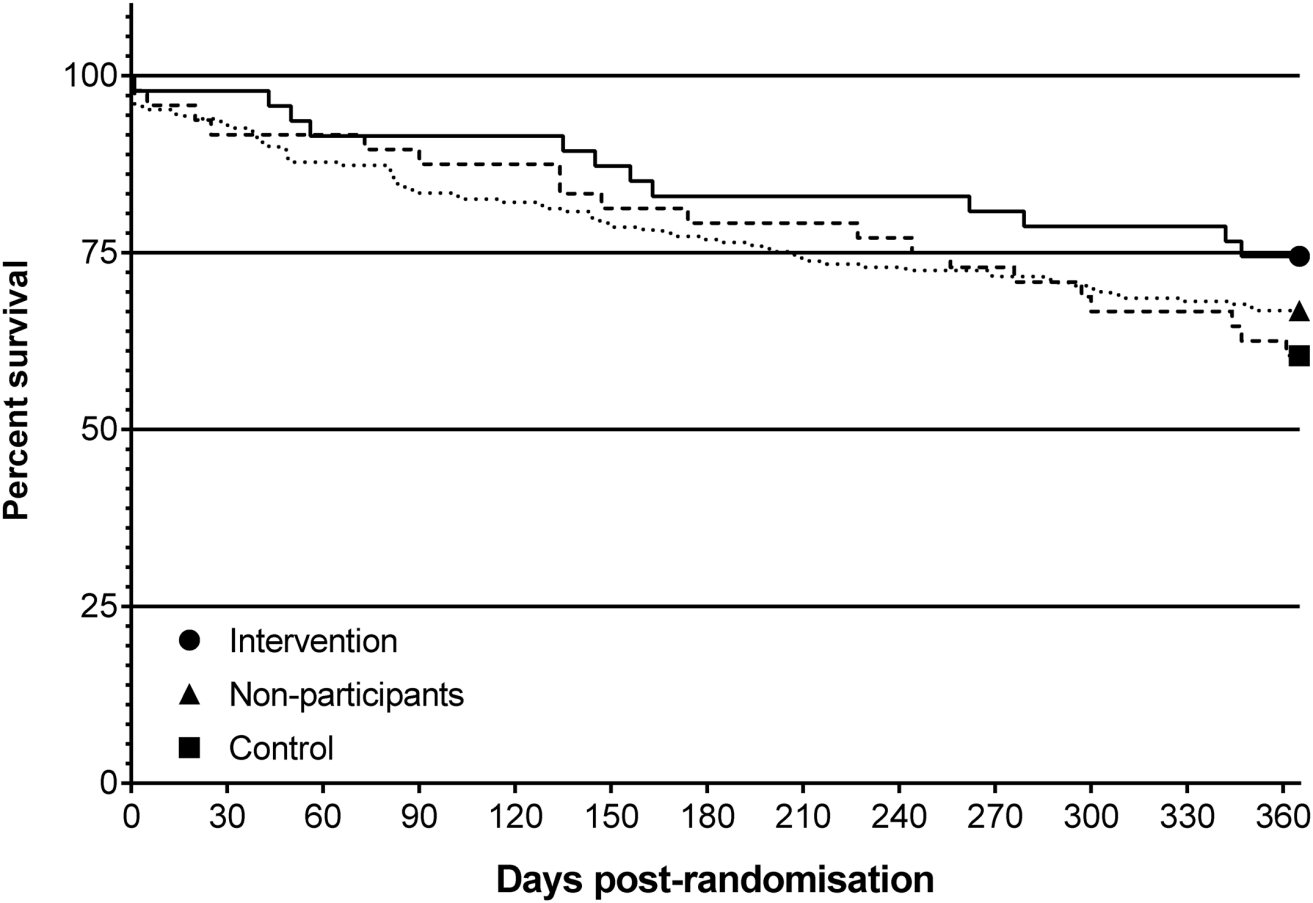
Kaplan Meier survival plot to 12 months post-randomisation.

Survival data are presented in Fig 5. In the twelve months from randomisation there were 12 deaths in the intervention group (26% mortality) and 19 deaths in the control group (40% mortality, χ^2^ 1.9, df 1, p = 0.16, HR 0.60, 95%CI 0.30 to 1.22)

## Discussion

Deprescribing reduced the number of regular medicines consumed by frail older people living in residential care in rural Western Australia. Risk-modifying medicines (aspirin, statins, antihypertensives, bisphosphonates, calcium, and vitamin D) were more successfully deprescribed than symptom-modifying medicines (analgesics, laxatives, antidepressants, hypnotics, and anxiolytics). The reduction in medicines at twelve months (two medicines per person) was half the number of medicines ceased during the trial (four medicines per person) and considerably smaller than the number of medicines selected for deprescribing (seven to eight medicines per person), confirming the difficulty of reducing polypharmacy in practice.

The main weakness of this study is the small sample size. We were unable to accurately estimate the effect of deprescribing on clinical outcomes other than the number of prescribed medicines or rule out adverse effects from the intervention. In addition, there were differences between groups at baseline in blood pressure and in medical diagnoses that may have affected the secondary outcomes, including survival rates.

The open design, while representing actual clinical practice, is also a major weakness, making it difficult to eliminate treatment biases or informal deprescribing in control group participants. Our protocol allowed GPs, RNs, and/or NOK to decline the cessation of any medicine in cognitively impaired participants. Although this might have hindered effective deprescribing, our data show that fewer than 4% of planned cessations were vetoed by a GP, nurse, or family member.

More than three-quarters of our study participants had dementia. Many of these people had difficulty reporting symptoms or adverse medicine withdrawal effects. We regularly checked progress notes and asked RACF staff about specific withdrawal symptoms, but we may have underestimated the negative effects of deprescribing in this population. People with advanced dementia were also unable to reliably complete sleep, quality of life and self-reported general health assessments. Our data on these outcomes are incomplete and unreliable. Using a proxy to assess these outcomes would have strengthened the data.

The main strength of this study is that we actively deprescribed rather than relying on indirect deprescribing methods such as recommendations to prescribers. The RACF setting meant we had accurate records of the medicines being consumed by our participants and we were able to confirm that deprescribed medicines were actually ceased. We minimised potential sources of bias by having a blinded research nurse assess the more subjective outcomes. Additional strengths are a randomised design with prospective registration of the protocol, per-protocol statistical analyses, and an appropriate control group treated as similarly as possible to the intervention group.

A 2008 systematic review of 31 studies that withdrew a single class of medicine in older people reported that diuretics, antihypertensives, benzodiazepines, and psychotropic agents could often be withdrawn without causing harm, but that psychotropics had a high rate of post-trial re-instatement.[43] High quality deprescribing studies that cease more than one class of medicine are rarer. Garfinkel et al. conducted two non-randomised deprescribing studies in a geriatric hospital and in community-dwelling older adults and achieved significant reductions in medicine use in both cohorts and significant improvements in survival and self-assessed general health respectively.[34,35] Gallagher et al. and Dalleur et al. conducted randomised studies using the Screening Tool of Older People’s Prescriptions (STOPP) to reduce the use of potentially inappropriate medicines (PIMs) in older hospital inpatients.[27,28,44] Gallagher et al. reported significantly reduced PIMs use in the intervention group at discharge and 6 months post-discharge, no change in the rate of hospital readmission, and non-significant reductions in falls, all cause-mortality, and GP visits during the 6 month follow-up period in 382 people aged 65 years and older.[27] Dalleur et al. enrolled frail inpatients aged over 75 years and reported reduced PIMs use in the intervention group on discharge, although the proportion of people prescribed at least one PIM was not altered.[28] Another recent randomised study investigated the effect of nurse training on potentially harmful medicine use in 227 residents of assisted living facilities in Helsinki and reported a small reduction in the use of potentially harmful medicines (-0.43, p = 0.004), fewer days in hospital, and a non-significant increase in mortality at 12 months in intervention group participants.[29]

Indirect deprescribing using education, medicine reviews, and advice to prescribers has been more frequently reported than direct deprescribing interventions where medicines are actively ceased by researchers. Indirect interventions achieve only small reductions in the number of medicines consumed by older people.[45] Consequently the impact of indirect interventions on clinical outcomes other than prescribing patterns is frequently insignificant or unreported. A systematic review and meta-analysis of the effect of pharmacist-led medication reviews on hospital admissions and mortality in older people reported a small reduction in the mean number of prescribed medicines (-0.48, 95% CI -0.89, -0.07) but no significant effect on hospital admissions (RR 0.99, 95% CI 0.87, 1.14, p = 0.92) or mortality (RR 0.96, 95%CI 0.82, 1.13, p = 0.65).[46]

Our results, when combined with evidence from these earlier studies, suggest that it may be possible to deprescribe in frail older people without adversely affecting survival or outcomes related to quality of life. We found that risk-modifying medicines with no symptomatic benefit were simple to deprescribe. We considered cessation of these medicines to be appropriate in people close to death where quality of life was a higher priority than extending survival. Our follow-up period was too short and participant numbers too small to determine whether discontinuation of these medicines would ultimately increase fracture and vascular event rates, but the theoretical risk of these events should be weighed against futility of treatment in people with very limited life expectancy and the potential for improved quality of life through reduced adverse drug effects and a reduced pill burden. Decision-making about symptom-modifying medicines was more difficult, particularly in people with cognitive impairment who were unable to reliably report symptoms. We were able to cease analgesics, laxatives, anti-reflux remedies, antidepressants, hypnotics, and anxiolytics without incident in 40% to 70% of people in whom withdrawal was attempted. These results suggest that withdrawal of symptom-modifying medicine is worth attempting if symptoms are stable and people are adequately monitored during and after deprescribing.

Quality deprescribing, as with good prescribing, requires the patient to be at the centre of the process. The risks of each medicine need to be weighed against the expected benefits in *this* specific person at *this* specific point in their life, taking into account their preferences and expectations, their likely prognosis, their co-morbidities, their symptoms, their other medicines, and the wishes and expectations of their family or carers. This is not a straightforward process and there is no simple list, guideline, or algorithm that will make it so. The protocol used in this study provides some guidance for doctors who wish to deprescribe. The results, while requiring confirmation in larger studies, suggest that careful deprescribing is unlikely to harm patients.

## Supporting Information

S1 CONSORT Checklist (DOCX)

S1 Protocol Research Protocol. (PDF)

S1 Table List of potential target medicines for deprescribing. (DOCX)

S2 Table Criteria for checking symptom stability when withdrawing and/or restarting medicines. (DOCX)

S3 Table Frequency of common medical diagnoses. (DOCX)

S4 Table Success rates for all medicines targeted for deprescribing. (XLSX)

S5 Table List of all new medicines in surviving participants at 12 months. (XLSX)
