# Supplementary material for: Deprescribing in Frail Older People: A Randomised Controlled Trial
Source: PLoS One. 2016 Mar 4;11(3):e0149984. doi: 10.1371/journal.pone.0149984 (PMC4778763; doi:10.1371/journal.pone.0149984)
Supplement: S1 Protocol — (PDF) [file pone.0149984.s002.pdf]

STUDY TITLE
Deprescribing in frail older people: a randomised controlled trial

AIMS
Our primary aim is to determine whether we can safely reduce the total number of medications prescribed to older people living in residential aged care facilities (RACF). Secondary aims are to estimate the effect of deprescribing on mortality, falls and fractures, sleep quality, cognitive function, and independence.

BACKGROUND
Pharmaceutical companies have a strong interest in encouraging doctors to prescribe and most research dollars are spent finding reasons to use drugs rather than reasons not to use them. Consequently, doctors receive a great deal of advice about starting medication but get very little guidance on the indications for stopping treatment. This is a significant problem. Older people are at high risk of adverse drug reactions and many medications cause confusion and falls in older patients (1, 2). In addition, evidence for the benefit of preventative and symptomatic therapies in frail older people is limited, as most clinical trials specifically exclude these patients (3, 4).

Over-prescribing is widely recognised as a serious problem in frail older people, but data on the effects of deprescribing are scarce. Several small observational studies and a few randomised trials have examined the effects of withdrawing a single class of medication in older people and report that antihypertensives, benzodiazepines and psychotropic agents can be stopped without harm (5). A recent Israeli trial reported the effect of reducing total medication burden in 190 disabled nursing-department patients (6). Garfinkel discontinued 332 different drugs in 119 patients (2.8 per person) and reported successful cessation of all target drugs in 82% of patients. The one-year mortality rate was 45% in the control group and 21% in the intervention group, a reduction in mortality of 53% ($p<0.001$). These data indicate that deprescribing is safe and achievable in frail older people and may reduce mortality. No randomised data on the effects of deprescribing have yet been published.

RESEARCH PROTOCOL

Study design
Randomised controlled intervention trial

Eligible participants
People aged 65 years and older living in RACF in Western Australia who consent to be randomised

Exclusion Criteria
Potential participants will be excluded from enrolment if they are:

(1) not taking any regular medication
(2) in the final terminal stages of cancer or other serious disease
(3) not competent to consent AND their next of kin does not agree to their participation
OR
(4) their usual doctor does not agree to their participation in the study

Recruitment
We will recruit participants from RACF in the mid-west region of Western Australia. There are a total of 261 high and low care RACF beds in this region. Geraldton has 221 beds divided between Geraldton Nursing Home (50 high care beds), Hillcrest (60 beds, 20 dementia and 40 low care) and Nazareth House (111 beds, mix of high and low care). The remaining 40 beds are divided between Meekathara (8 beds), Northampton (8 beds), Dongara (6 beds), Three Springs (8 beds), and Morawa (10 beds). We will enrol as many residents of these facilities as possible. If necessary, we will also recruit in RACF in Perth to reach our target sample size of 250 participants.

Establishing informed consent
The difficulty of establishing informed consent in frail older people, particularly those with cognitive impairment, has often been used to exclude them from clinical trials. Consequently evidence for the efficacy and benefit of many symptomatic and preventative treatments in this population is lacking, while there is ample evidence of the dangers of over-medication. We believe that frail older people have the right to participate in research should they wish to. We have thus planned our consent process with care, to allow our target population the opportunity to participate in research while safeguarding their interests by involving their next-of-kin and usual doctor in the consent process.

We will seek written permission from RACF managers and corporate owners to recruit in their facilities. We will inform GPs who care for the residents about the study and ask for permission to recruit their patients. We will approach potential participants directly, explain our study aims and provide a participant information form (PIF). Any patient who declines to participate will not be enrolled in the study. Cognition will be assessed with the Mini-Mental Status Examination (MMSE) in individuals who agree to participate. Participants with a MMSE of 24 or greater will be considered competent to formally consent to inclusion in the study. If these participants agree, we will also inform their nominated next-of-kin (NOK) of their desire to participate in our project and provide the NOK with a study information sheet. If a participant wishes to participate in the study, but is not competent to formally consent (MMSE less than 24), we will request written agreement to their participation from their nominated next-of-kin. When we have gained written agreement from participants or their next-of-kin, we will ask their GP to also provide written assent to their randomisation. We will give the GP a list of all their patients who have consented to participate and ask them to approve the list.

Intervention
Sequential withdrawal of medication according to a structured deprescribing protocol (Figure 1)

Primary outcome
The total number of medications taken by participants at 12 months post-enrolment

Secondary outcomes

- Mortality at 12 months post-enrolment
- Falls and non-vertebral fractures at 12 months post-enrolment
- Sleep quality, assessed by the Neuropsychiatric Inventory–Nursing Home version (NPI-NH) at 3, 6, and 12 months post-enrolment
- Cognitive function, assessed by the mini-mental state examination (MMSE) at 3, 6, and 12 months post-enrolment
- Independence in activities of daily living, assessed by the modified Barthel index at 3, 6, and 12 months post-enrolment
- Number of medications taken by participants at 3 and 6 months post-enrolment

Baseline data
We will record a detailed medical history and conduct a clinical examination of each participant. We will record the generic name, indication, dosage, and frequency of all medications taken by the participant, including all prescribed and over-the-counter medications and any herbal or mineral supplements. We will determine the indication for each medication by discussion with the participant, the participant's doctor, and, if necessary, by accessing the participant's medical records. We will administer the MMSE, NPI-NH and the modified Barthel index and measure weight and blood pressure.

Randomisation
Participants will be randomly allocated to intervention or control group by block randomisation.

Identification of target drugs for withdrawal
Two investigators will independently review the medication lists and identify target drugs for withdrawal based on previously published guidelines (Table 1) and the flow chart below (6, 8-11). Both investigators will independently determine the order of drug withdrawal. Drugs least likely to cause an adverse drug withdrawal event (ADWE) will be ceased before drugs most likely to cause an ADWE. The investigators will resolve by consensus any differences in the target drugs and withdrawal order.

Table 1. Potential target medications for withdrawal
(Based on previously published guidelines for prescribing in older patients (6, 8-11))

	**Inappropriate medications**	**Medications with no symptomatic benefit**	**Medications with possible symptomatic benefit**
**Adverse drug withdrawal event likely**	Benzodiazepines, antipsychotics, tricyclic antidepressants, long-acting sulphonylureas	Antihypertensives	Opioid analgesics, inhaled and oral corticosteroids, diuretics, antiemetics, oral and topical oestrogens, digoxin
**Adverse drug withdrawal event less likely**	NSAIDs, antispasmodics, anticholinergic antihistamines, short-acting calcium channel blockers, stimulant laxatives, muscle relaxants, dipyridamole, nitrofurantoin, ditropan, amiodarone	Statins, potassium supplements, mineral supplements, vitamins	Nitrates, antacids, antireflux medications, iron supplements, herbal remedies, cough suppressants, nasal decongestants

Figure 1. Deprescribing flowchart
(Modified from Garfinkel and Mangin (7))

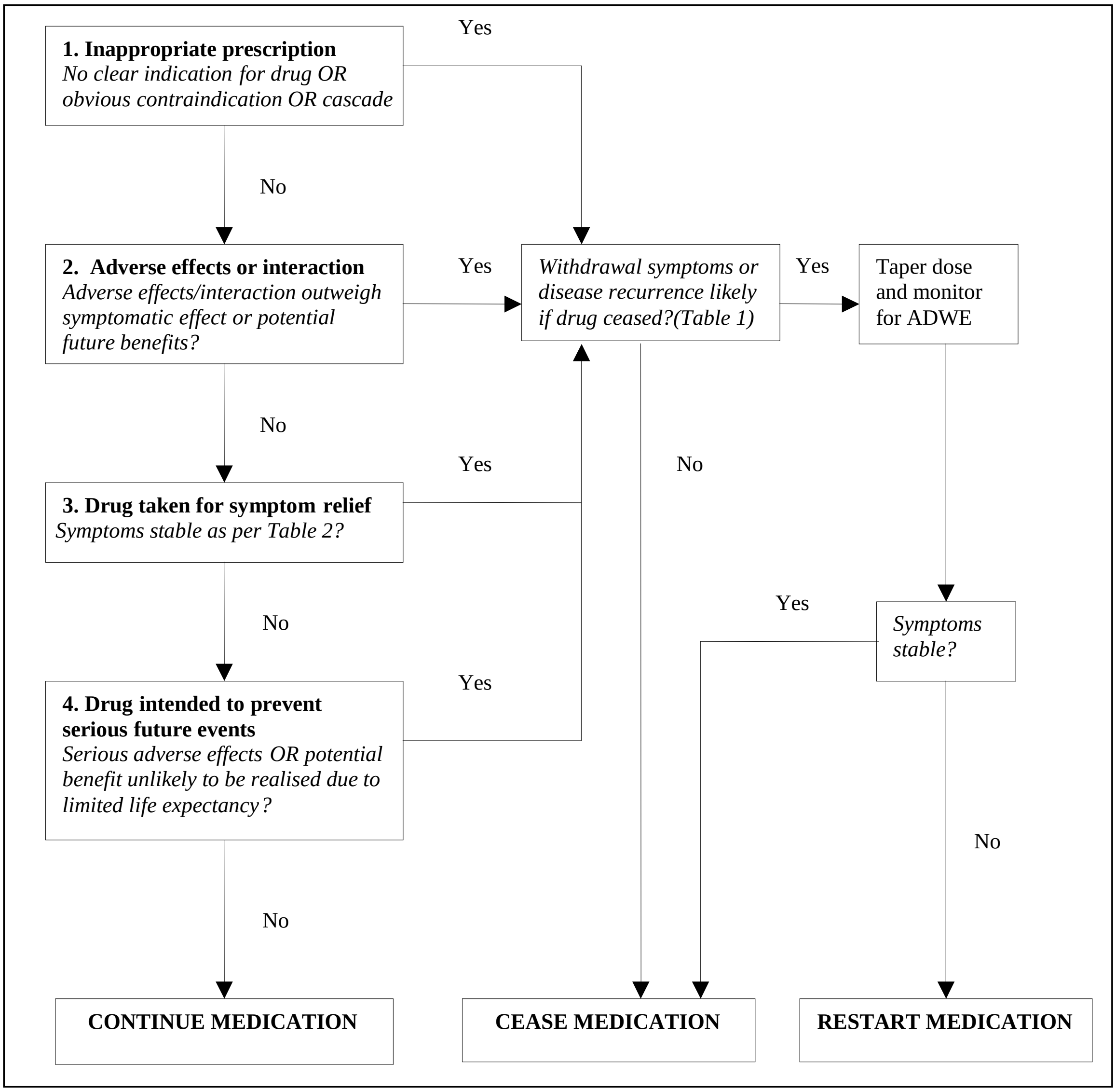

Intervention group

We will advise intervention group participants' doctors that their patient has been randomised to the intervention group. We will offer to conduct the drug withdrawal ourselves. If participants' doctors do not want us to conduct the drug withdrawal and would prefer to manage it themselves, we will provide a list of the target medications, a withdrawal protocol, a table of indications for restarting withdrawn medications, and regular reminders about target medications that have not been ceased. If the participant's doctor is happy for us to conduct the drug withdrawal, we will cease or reduce the dose of the first target medication. Prior to ceasing or reducing the dose of each medication, we will inform nursing staff of the common adverse withdrawal effects associated with that drug. The nurse manager will have the mobile phone number of the chief investigator and will be encouraged to contact her if there are any concerns about adverse withdrawal effects in any study participant. Participants will be reviewed twice weekly for ADWE after cessation or dose reduction of the first target medication. If symptoms are stable according to pre-defined criteria (Table 2) and no ADWE is reported after two weeks, we will further reduce the dose of the first target drug or withdraw the next target medication. We will review the participant twice weekly for a further two weeks and, if symptoms are stable, cease or reduce the dose of the next target medication and so forth. Participants will be reviewed twice weekly until all target medications are withdrawn and

symptoms are stable. We will then review the participant weekly for a further two visits and, if stable, return them to the care of their usual doctor.

All regular medications will be administered by Webster-pak™ and we will communicate each change in medication to the dispensing pharmacist and usual treating doctor. Where possible dose reductions will be achieved by cutting tablets in half or quarters to avoid extra dispensing and prescription costs to participants. Pro re nata medications will continue to be administered by the nursing staff or patient, according to the protocol of the individual aged-care facility. We will record all drug withdrawal failures and the reasons for failure.

Table 2. Indications for withdrawal and re-instatement of specific target medications

Condition	Target medication	Withdraw medication if:	Restart medication if:
Ischaemic heart disease	Long-acting nitrates	No chest pain in the previous 6 months	Recurrence of chest pain or SOB on exertion or at rest.
Gastro-oesophageal reflux disease	Antacids, H2 blockers, PPIs	No proven peptic ulcer and no gastrointestinal bleeding for 1 year. No dyspepsia for 6 months.	Recurrence of dyspepsia or other gastrointestinal symptoms attributable to withdrawal of treatment
Heart failure	Diuretics	No orthopnea or peripheral oedema for last 6 months	Recurrence of peripheral oedema, dyspnoea or orthopnea, or >2.5kg weight gain
Constipation	Stimulant laxatives	Regular bowel movements for last 6 months	Failure to open bowels for more than 72 hours
Chronic obstructive airways disease	Inhaled or oral corticosteroids	Steroid responsiveness has not been established or stable symptoms last 6 months	Worsening dyspnoea
Nausea	Antiemetics	Asymptomatic with no nausea or vomiting for more than 3 months	Recurrence of nausea or vomiting
Vertigo	Anti-dizziness medications	Asymptomatic with no episodes of dizziness for more than 3 months	Recurrence of symptoms or a fall
Hypertension	Anti-hypertensives	BP <160/90	Increase in BP above 160mmHg systolic or 90mmHg diastolic
Hypokalaemia	Potassium supplements	Normal serum potassium	Potassium < 3.0mmol/L
Iron deficiency	Iron supplements	Normal Hb, serum iron and ferritin levels and no known reason for iron deficiency	Hb < 100 AND serum iron < 7μmol/L OR ferritin <30 pmol/L
Depression	Anti-depressants	Stable mood, sleep and appetite for previous 6 months	Recurrence of mood symptoms, change in appetite or sleep disturbance
Diabetes	Long-acting oral hypoglycaemic agents, glitazone	HbA1c <8%, stable BSL for previous 6 months	Polyuria, fasting BSL >15 OR HbA1c > 10% at 6 weeks after withdrawal
Atrial fibrillation	Amiodarone	On a rate-controlling medication AND not in sinus rhythm	Symptomatic tachycardia
Urge incontinence	Anticholinergics	Cognitive impairment OR resident now managed with containment	Symptomatic urgency recurs

Control group
We will advise control group participants' doctors that their patient has been randomised to the control group. We will visit control participants with same frequency as we visit intervention group participants during the drug withdrawal phase of the trial. We will measure blood pressure at each visit and make general enquiries as to any problems the participant may wish to report. At the end of the 12-month trial period, we will provide the usual treating doctor with a list of target medications, withdrawal protocols, and a list of indications for restarting withdrawn medications.

Follow-up data
We will follow-up all participants at three, six, and twelve-months post-enrolment. We will record all medications, deaths, falls and non-vertebral fractures. We will reassess MMSE, NPI-NH, and the modified Barthel index, and measure weight and sitting blood pressure.

Data safety monitoring
Any adverse or serious adverse events in the intervention or control group that occur during the drug withdrawal phase of the trial or during the follow-up period will be recorded on the Serious Adverse Event Report form and forwarded to a data safety monitoring board. The board is comprised of an independent clinical expert and a statistician. Neither of these individuals will be involved with data collection or data analysis. Serious adverse events are defined as death, hospital admission, fall, non-vertebral fracture, non-fatal vascular event (MI or CVA), and after-hours GP attendance. The nurse managers of the homes will be asked to advise the investigator as soon as practical if any of these events occur in study participants. The data safety monitoring board will convene three-monthly during the study period to compare adverse event rates in the intervention and control groups. We will consider ceasing the trial early if the differences between the groups in adverse events reach a p-value of 0.01 and the difference appears to be due to the study intervention.

Sample Size
We will enrol 250 participants. We will have power to detect a difference between groups in the mean number of medications of 1.34 (α=0.05 and 1-β =0.8, SD 3.9). Garfinkel achieved a mean reduction of 2.8 medications per subject and reported 53% reduction in mortality at one year(6). West Australians with a diagnosis of dementia living in RACF have a 25% one-year mortality (DIRECT, unpublished data). The contingency table below shows the sample size needed per group to detect the indicated change in one-year mortality for α=0.05 and 1-β =0.8, assuming a baseline mortality of 25%. A sample size of 250 people will give us adequate power to detect a very large change in mortality but not to exclude a moderate effect. This proof-of-concept trial will allow us to determine absolute event rates in the intervention and control groups and to calculate more precise recruitment targets for a definitive deprescribing trial with mortality as the primary outcome.

Mortality rate in intervention group	Change in mortality in intervention group	Number per group
0.40	+60%	165
0.35	+40%	349
0.30	+20%	1291
0.20	-20%	1134
0.15	-40%	270
0.10	-60%	113

Statistical Analyses

Data will be analysed on an intention-to-treat basis. We will compare the number of medications at one year with the Wilcoxon rank-sum test. We will assess one-year mortality with Fisher's exact test. We will also use Kaplan-Meier survival curves and the Cox proportional hazards model to compare survival in the intervention and control groups. We will use Fisher's exact test to compare the proportion of participants in each group that have experienced a fall or fracture at six months and one year post-enrolment. We will compare MMSE, NPI-NH, and the modified Barthel index scores at three, six and twelve months using a generalised linear model adjusted for baseline scores, age, sex, and total number of prescribed medications at baseline. P-values of less than 0.05 will be considered significant.

SIGNIFICANCE OF PROPOSAL

The number of older people in Australia is increasing. More than 13% of Australians are aged 65 years or over. Within fifty years this group will represent 25% of the population and up to 7% of people will be aged 85 years or over (12). Medication use increases with age and people living in RACF are prescribed more medications than people of similar age living in the community. People with dementia living in RACF in WA take 9.8 ± 3.9 medications(13) and approximately 20% of these patients are prescribed at least one inappropriate medication (14).

Our study will be the first randomised trial to investigate deprescribing. If our data show that medication burden can be reduced safely in frail older people living in RACF, this project will support a large definitive deprescribing trial powered to detect clinically important effects on mortality. If deprescribing is shown to be beneficial, the target population will benefit from a reduced medication burden, improved quality of life, and lower pharmaceutical costs. The financial benefits to the Australian government may also be substantial.

REFERENCES

1. Schwartz J, Abernethy D. Aging and medications: Past, present, future. Clinical Pharmacology and Therapeutics 2009;85(1):3-10.
2. Le Couteur DG, Hilmer SN, Glasgow N, Naganathan V, Cumming RG. Prescribing in older people. Aust Fam Physician 2004;33(10):777-81.
3. Hilmer SN, Mager DE, Simonsick EM, Cao Y, Ling SM, Windham BG, et al. A drug burden index to define the functional burden of medications in older people. Arch Intern Med 2007;167(8):781-7.
4. Holmes HM, Hayley DC, Alexander GC, Sachs GA. Reconsidering medication appropriateness for patients late in life. Arch Intern Med 2006;166(6):605-9.
5. Iyer S, Naganathan V, McLachlan AJ, Le Conteur DG. Medication withdrawal trials in people aged 65 years and older. Drugs & Aging 2008;25(12):1021-1031.
6. Garfinkel D, Zur-Gil S, Ben-Israel J. The war against polypharmacy: a new cost-effective geriatric-palliative approach for improving drug therapy in disabled elderly people. Isr Med Assoc J 2007;9(6):430-4.
7. Garfinkel D, Mangin D. Feasibility study of a systematic approach for discontinuation of multiple medications in older adults: addressing polypharmacy. Arch Intern Med 2010;170(18):1648-1654.
8. Fick DM, Cooper JW, Wade WE, Waller JL, Maclean JR, Beers MH. Updating the Beers criteria for potentially inappropriate medication use in older adults: Results of a US consensus panel of experts. Arch Intern Med 2003;163(22):2716-2724.
9. Laroche ML, Charmes JP, Merle L. Potentially inappropriate medications in the elderly: a French consensus panel list. Eur J Clin Pharmacol 2007;63(8):725-31.
10. Basger BJ, Chen TF, Moles RJ. Inappropriate medication use and prescribing indicators in elderly Australians: Development of a prescribing indicators tool. Drugs & Aging 2008;25(9):777-793.
11. Woodward MC. Deprescribing: Achieving better health outcomes for older people through reducing medications. Geriatric Therapeutics 2003.
12. Statistics ABo. 3222.0 - Population Projections, Australia, 2006 to 2101. In; 2008.
13. Somers M, Simmonds D, Rose E, Whitelaw C, Calver J, Beer C. Quality use of medicines in residential aged care. Australian Family Physician 2010;In Press(Accepted February 2010).
14. King M, Roberts M. The influence of the Pharmaceutical Benefits Scheme (PBS) on inappropriate prescribing in Australian nursing homes. Pharmacy World & Science 2007;29(1):39-42.
