# Supplementary material for: Deprescribing in Frail Older People: A Randomised Controlled Trial
Source: PLoS One. 2016 Mar 4;11(3):e0149984. doi: 10.1371/journal.pone.0149984 (PMC4778763; doi:10.1371/journal.pone.0149984)
Supplement: S1 Table — (DOCX) [file pone.0149984.s003.docx]

| **Inappropriate Medicines** | **Medicines with no symptomatic benefit** | **Medicine with symptomatic benefit** |
| --- | --- | --- |
| Benzodiazepines, antipsychotics, tricyclic antidepressants, long-acting sulphonylureas,  NSAIDs, antispasmodics, anticholinergic antihistamines, short-acting calcium channel blockers, stimulant laxatives, muscle relaxants, dipyridamole, nitrofurantoin, ditropan, amiodarone | Antihypertensives,  Statins, potassium supplements, mineral supplements, vitamins | Opioid analgesics, inhaled and oral corticosteroids, diuretics, antiemetics, oral and topical oestrogens, digoxin, nitrates, antacids, anti-reflux remedies, iron supplements, herbal remedies, cough suppressants, nasal decongestants |

**S1 Table: List of potential target medicines for deprescribing**
