# Supplementary material for: Deprescribing in Frail Older People: A Randomised Controlled Trial
Source: PLoS One. 2016 Mar 4;11(3):e0149984. doi: 10.1371/journal.pone.0149984 (PMC4778763; doi:10.1371/journal.pone.0149984)
Supplement: S2 Table — (DOCX) [file pone.0149984.s004.docx]

| **Condition** | **Target medication** | **Withdraw medication if:** | **Restart medication if:** |
| --- | --- | --- | --- |
| Ischaemic heart disease | Long-acting nitrates | No chest pain in the previous 6 months | Recurrence of chest pain or SOB on exertion or at rest. |
| Gastro-oesophageal reflux disease | Antacids, H2 blockers, PPIs | No proven peptic ulcer and no gastrointestinal bleeding for 1 year. No dyspepsia for 6 months. | Recurrence of dyspepsia or other gastrointestinal symptoms attributable to withdrawal of treatment |
| Heart failure | Diuretics | No orthopnea or peripheral oedema for last 6 months | Recurrence of peripheral oedema, dyspnoea or orthopnea, or >2.5kg weight gain |
| Constipation | Stimulant laxatives | Regular bowel movements for last 6 months | Failure to open bowels for more than 72 hours |
| Chronic obstructive airways disease | Inhaled or oral corticosteroids | Steroid responsiveness has not been established or stable symptoms last 6 months | Worsening dyspnoea |
| Nausea | Antiemetics | Asymptomatic with no nausea or vomiting for more than 3 months | Recurrence of nausea or vomiting |
| Vertigo | Anti-dizziness medications | Asymptomatic with no episodes of dizziness for more than 3 months | Recurrence of symptoms or a fall |
| Hypertension | Anti-hypertensives | BP <160/90 | Increase in BP above 160mmHg systolic or 90mmHg diastolic |
| Hypokalaemia | Potassium supplements | Normal serum potassium | Potassium < 3.0mmol/L |
| Iron deficiency | Iron supplements | Normal Hb, serum iron and ferritin levels and no known reason for iron deficiency | Hb < 100 AND serum iron < 7μmol/L OR ferritin <30 pmol/L |
| Depression | Anti-depressants | Stable mood, sleep and appetite for previous 6 months | Recurrence of mood symptoms, change in appetite or sleep disturbance |
| Diabetes | Long-acting oral hypoglycaemic agents, glitazone | HbA1c <8%, stable BSL for previous 6 months | Polyuria, fasting BSL >15 OR HbA1c > 10% at 6 weeks after withdrawal |
| Atrial fibrillation | Amiodarone | On a rate-controlling medication AND not in sinus rhythm | Symptomatic tachycardia |
| Urge incontinence | Anticholinergics | Cognitive impairment OR resident now managed with containment | Symptomatic urgency recurs |

###### S2 Table: Criteria for checking symptom stability when withdrawing and/or restarting medicines
