# Supplementary material for: Deprescribing in Frail Older People: A Randomised Controlled Trial
Source: PLoS One. 2016 Mar 4;11(3):e0149984. doi: 10.1371/journal.pone.0149984 (PMC4778763; doi:10.1371/journal.pone.0149984)
Supplement: S3 Table — (DOCX) [file pone.0149984.s005.docx]

| **Diagnosis** | **Intervention (n=47)** | **%** | **Control (n=48)** | **%** |
| --- | --- | --- | --- | --- |
| Hypertension | 30 | 64 | 32 | 67 |
| Osteoarthritis | 25 | 53 | 28 | 58 |
| Depression | 20 | 43 | 28 | 58 |
| GORD | 17 | 36 | 28 | 58 |
| Fracture | 16 | 34 | 8 | 17 |
| Cerebrovascular event | 11 | 23 | 6 | 13 |
| Hypothyroidism | 9 | 19 | 9 | 19 |
| Asthma/COPD | 8 | 17 | 10 | 21 |
| Atrial Fibrillation | 8 | 17 | 11 | 23 |
| Chronic kidney disease | 8 | 17 | 2 | 4 |
| NIDDM | 8 | 17 | 9 | 19 |
| Cancer | 7 | 15 | 8 | 17 |
| Congestive cardiac failure | 7 | 15 | 9 | 19 |
| Parkinsonism | 7 | 15 | 7 | 15 |
| Anxiety | 6 | 13 | 8 | 17 |
| Ischemic heart disease | 6 | 13 | 14 | 29 |
| Epilepsy/seizures | 5 | 11 | 2 | 4 |
| Glaucoma | 5 | 11 | 4 | 8 |
| Gout | 3 | 6 | 4 | 8 |

**S3 Table: Frequency of medical diagnoses**
